# Effectiveness of intravaginal electrical stimulation combined with electromyography biofeedback-mediated pelvic floor muscle training for postpartum symptomatic pelvic organ prolapse: protocol for the PROSPECT randomized trial

**DOI:** 10.1186/s13063-022-06051-z

**Published:** 2022-02-09

**Authors:** Li Min, Yang Chunxue, Lv Qiubo, Dong Xudong, Zhang Yan, Zhang Guifang, Hu Kejia, Gai Tianzi, Feng Qing

**Affiliations:** 1 Department of Obstetrics and Gynecology, Beijing Hospital, National Center of Gerontology, Institute of Geriatric Medicine, Chinese Academy of Medical Sciences, No. 1 Dahua Road, Dongdan, Dongcheng District, Beijing, 100730 China; 2 Peking University Health Science Center, Beijing, 100069 China; 3 Department of Obstetrics, the First People’s Hospital of Yunnan Province, Affiliated Hospital of Kunming University of Science and Technology, Kunming, 650032 Yunnan China; 4 Department of Obstetrics and Gynecology, Beijing Chaoyang Maternal and Child Health Hospital, Beijing, 100026 China; 5 The Key Laboratory of Geriatrics, Beijing Institute of Geriatrics, Beijing Hospital, National Center of Gerontology, National Health Commission; Institute of Geriatric Medicine, Chinese Academy of Medical Sciences, Beijing, 100730 China; 6 Department of Obstetrics and Gynecology, Beijing Shunyi Traditional Chinese Medicine Hospital, Beijing, 101300 China

**Keywords:** Prolapse, Pelvic floor muscle, Biofeedback, Electrical stimulation, Quality of life, Economic evaluation

## Abstract

**Background:**

Pelvic organ prolapse (POP) is seen in up to 30–70% of women presenting for routine gynecology care and 10% of women suffering from bothersome POP symptoms. Vaginal childbirth is one of the most prominent contributing factors for POP. Pelvic muscle training (PFMT) is considered effective to improve mild to moderate POP symptoms. In addition, higher-intensity, supervised PFMT aided by biofeedback and electrical stimulation may confer greater benefit. However, the long-term efficacy of the PFMT combined with electromyography biofeedback is unknown, which indicates the need for further evidence.

**Methods:**

This multicenter randomized controlled trial compares the effectiveness and cost-effectiveness of conventional PFMT versus biofeedback-mediated PFMT plus neuromuscular electrical stimulation (NMES) for postpartum symptomatic POP women. The primary outcome is the proportion of patients with the improvement of at least one POP-Q stage at 36 months after randomization. The primary economic outcome measure is incremental cost per quality-adjusted life year at 36 months. Two hundred seventy-four women from four outpatient medical centers are randomized and followed up with pelvic floor examination, questionnaires, and pelvic ultrasonography imaging. All participants are arranged for three appointments over 12 weeks. NMES and electromyography biofeedback via intravaginal probe are added to PFMT for participants in the biofeedback-mediated PFMT group. Group allocation could not be blinded from participants and healthcare staff that deliver intervention but remain masked from medical staff that carry out PFM assessment. An intention-to-treat analysis of the primary outcome will estimate the difference of the proportion of POP-Q stage improvement between the trial groups right after the intervention, and during the follow-up until 36 months, using the chi-square test and multilevel mixed models respectively. A logistic regression analysis was used for adjusting for important baseline variables that are unbalanced.

**Discussion:**

The trial results will provide evidence of the effectiveness of electromyography biofeedback-mediated PFMT in improving women’s POP-Q staging, quality of life, and cost-effectiveness.

**Trial registration:**

CCTR Registry ChiCTR210002171917. Registered on March 6, 2019

## Background and rationale {6a}

Pelvic organ prolapse (POP), encompassed in the spectrum of pelvic floor dysfunction (PFD), is a common condition characterized by a descent of the female pelvic organs (bladder, uterus, and rectum) from the normal anatomic position into or through the vagina [1]. Half parous women have varying degrees of POP, and one in 12 women suffered from bothersome POP symptoms [2] which negatively influence women’s social, physical, and psychological well-being [3]. Direct medical cost for PFDs is estimated to total $412 million for ambulatory care and $1012 million for POP surgery annually in the USA [4]. The number of patients in need of POP treatment is on a rapid rise due to an aging population and an improving awareness [5]. Treatment for POP includes conservative options (like pelvic floor muscle training, PFMT) and surgery. The former is recommended for all POP patients and particularly helpful for those with a mild to moderate degree of prolapse [6–8], while the latter is reserved for those having severe symptomatic prolapse with the goal of anatomical restoration and symptom relief [1]. Given the substantial societal and economic burden, there is a growing demand to identify and validate more tailored and effective treatment strategies addressing the pathogenesis of POP.

POP’s most consistent risk factors include vaginal delivery, previous hysterectomy, menopausal state, high body mass index, and genetic background [5], all of which result in weakened pelvic floor musculature and connective constructure. With direct damage to the fetal passage, vaginal delivery is one of the most prominent contributing factors to POP. Delancey et al. [9] reported that childbirth-induced injury of levator ani presented in 55% of women with prolapse, yet only 16% in those with normal support. Reportedly 56% primiparous and 58.8% multiparous women manifest different levels of PFM strength impairment early postpartum [10, 11], paralleled with the time POP commonly develops. The incidence rate of stage 2–4 prolapse (determined by the Pelvic Organ Prolapse Quantification System, POP-Q) during this period is estimated as high as 64% among primiparous women [12]. Early postpartum intervention has been reported to ameliorate PFM morphology and function. Patients that initiated PFMT 6 weeks after delivery demonstrated increased PFM strength and endurance. Further stratum of those with a major defect of levator ani muscle showed 45% less chance of having vaginal symptoms in the PFMT group [13–15]. However, a systemic review [16] identified little benefit of postnatal PFMT in treating urinary and fecal incontinence more than 3 months postpartum, presumably attributed to a spontaneous discontinuation of PFMT and an increasing burden on the pelvic floor which resulted from holding the growing newborn. Follow-up data beyond the first postpartum year is extremely rare. High-quality evidence evaluating postnatal PFMT remains a necessity.

PFMT improves strength, endurance, coordination, and timing of PFM contraction; facilitates appropriate muscle tone during relaxation; and hypertrophies the pelvic floor, thereby aiding in structural support [17–20]. However, when executing a conventional PFMT, more than 30% of women are unaware what muscles they have actually recruited when voluntarily training themselves, and also what muscles they are supposed to activate and how for an effective contraction [21, 22], which partially account for the mixed results of the efficacy of PFMT in individual studies. That is where neuromuscular electrical stimulation (NMES) and electromyography biofeedback kick in as an adjunct. NMES stimulates PFM activity in a similar way our nerve system does, except that lack of motivation and fatigue may jeopardize our adherence to voluntarily, sufficiently, and continually exercising our PFM, whereas an NMES program automatically turns PFMT into a supervised routine [23]. Meanwhile, signals from our nervous system may not pick up enough fibers, as nerves and neuromuscular junctions are stretched, compressed, and frequently impaired along pregnancy progression and when babies push through [24], while external signals from the NMES device might hopefully be capable of. Electrical impulses generated by an NMES device are delivered through electrodes bedded on an intravaginal probe or under a skin pad to targeted nerves and muscle fibers to elicit PFM contraction [21, 25]. Within EMG biofeedback, EMG delineating the superposition of electrical potentials arising from activated muscle fibers is collected via electrodes and processed into visual and auditory guidance, leading patients to a correct contraction. Targeted signals enable selective training and thus strengthening of individual muscles [26]. Visualization of contraction and responsive instruction add more interaction to basic PFMT, potentially promoting practice initiative and body awareness.

Despite that NMES has been trialed and reviewed for years in verification of its theoretical advantages [27–29], few studies have featured the real-world effect of NMES and biofeedback combined PFMT on postpartum POP, and they generally assessed treatment response symptom-wise. Therefore, we will supplement our trial with more objective measurements, including the morphology and electrophysiology of PFM, the anatomical staging of POP, and the cost analysis. In combination, they are intended to test our hypothesis that NMES combined PFMT may have a superior efficacy on POP with better modulation of intrinsic PFM. This approach excels for it digs deeper to the root of POP.

### Objectives {7}

In this study, we aim to determine whether PFMT assisted by NMES and EMG biofeedback, in comparison to conventional PFMT, brings forth more satisfactory short-term and long-term changes anatomically and clinically among early postpartum POP patients and to compare the cost-effectiveness of both training programs.

## Methods

### Trial design {8}

**Fig. 1:**
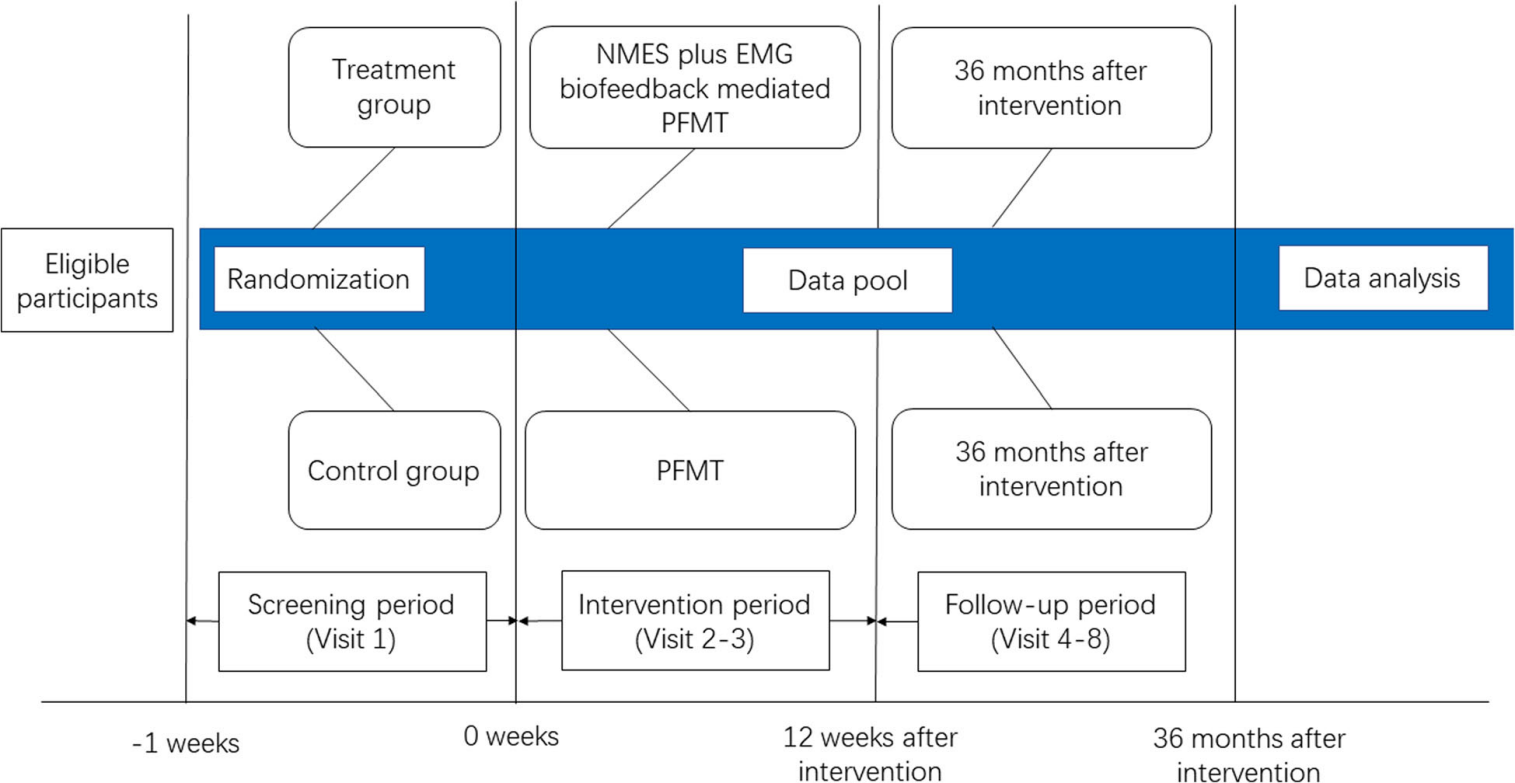
Flow chart of PROSPECT study

PROSPECT (to treat *pro*lapse *s*uffered *p*ostpartum with *e*lectrical stimulation-*c*ombined *t*raining of pelvic floor muscle) study comprises a two-armed, multicenter randomized controlled trial (Fig. 1). We anticipate to recruit 274 eligible primiparas in the 6th postpartum weeks and randomly assign them (by sealed envelope) on a 1:1 ratio into an experimental group that receives NMES and EMG biofeedback-mediated PFMT and a control group that receives conventional PFMT alone. Group allocation could not be blinded from participants. Within a 3-year follow-up, we will continuously monitor the effect on POP by measuring:

- Commonly mentioned POP-related symptoms with questionnaires
- Anatomic position of pelvic organs and morphology of pelvic floor through POP-Q staging and ultrasonography
- PFM strength by digital palpation under the Modified Oxford Scale
- PFM electromyography employing the Glazer Protocol

The cost-effectiveness of NMES and EMG biofeedback combined PFMT will be established by comparing direct medical costs (costs for outpatient consultations, treatment devices usage, absorbent pads, and surgery when required) from hospital documentation and participant-completed questionnaires.

### Methods: participants, interventions, and outcomes

#### Study setting {9}

The RCT will simultaneously take place in 4 centers spanning northeast and southwest China, namely Beijing Hospital, Beijing Friendship Hospital, Beijing Dongcheng District Health and Family Planning Service Center, and First People’s Hospital of Yunnan Province. All of them are academic hospitals equipped with NMES devices and well-trained staff specializing in pelvic floor rehabilitation. Registration information will be available on http://www.chictr.org.cn, the official website of Chinese Clinical Trial Registry, a non-profitable organization that has gained membership in WHO ICTRP. An internal pilot initiated in Beijing Hospital is intended to help us accumulate experience and optimize our design for this RCT.

#### Eligibility criteria {10}

##### Inclusion and exclusion criteria for participants

Women meet the inclusion criteria if they are primiparas between 20 and 40 years of age, at the 6th to 8th week after delivering a singleton baby vaginally full-term and non-instrumented, without a vaginal laceration over grade 3, with anatomical evidence of mild to moderate prolapse (2 ≤ POP-Q stage ≤ 3). We excluded women with factors that may confound the evaluation of impacts on pelvic floor function, such as a chronic cough or constipation, POP or UI history prior to pregnancy, previous pelvic surgery, or induced abortion in the mid-trimester. Non-Mandarin speakers and those under conditions like mental disorders or illiteracy are also excluded, as their decision-making and instruction-grasping abilities are impaired. Also considered unqualified for this study are those with contraindications for NMES, including a pelvic malignancy, a genitourinary infection, or an implanted pacemaker.

##### Eligibility criteria for healthcare providers

Therapists involved in this study are experienced and certificated in pelvic floor rehabilitation, all acquainted with the manipulation of relevant devices. They will receive additional training prior to the start of the study. A manual with standard operation protocol (SOP) will be provided to each center covering details on the execution of all procedures adopted in this study. The same equipment and SOP will be employed across centers for standardization.

#### Recruitment {15}

A postnatal consultation is normally scheduled at the 6th week after delivery in China, during which gestation and childbirth history, POP-Q staging, and digital palpation are routinely taken. All prolapse, structural defects, painful areas on palpation, POP-related symptoms, and functional assessments of PFM are documented. Upon screening of those clinical records, primiparas aged 20–40 with stage 2–3 (in POP-Q system) prolapse who have had full-term non-instrumented vaginal delivery are identified as potential participants for this study. They are referred to a recruitment advertisement with detailed information on study layout and eligibility. If showing interest, they will be given a pamphlet and several days to adequately understand the research. Later on, we will approach them by phone and invite them to a research center in an appointment that is termed the baseline visit.

#### Who will take informed consent? {26a}

A principal investigator is designated in each research center to implement the informed consent process throughout the entire time scope of the study. After confirming the inclusion eligibility of potential participants in the baseline visit (see the above section), the investigator will present to them the panorama of this study again, with an emphasis on putative benefits and risks of each intervention and scheduled follow-up visits as well as examinations to be performed during them. The investigator needs to make sure they have thoroughly weighed their decision and that they understand the voluntary nature of their participation where dropout at any point is permitted without punishment. They are notified on the confidentiality of their identity information and the privacy of data management. Written consent is asked if they readily wish to enroll after being fully informed. No study procedures are allowed until the consent has been taken. Participants are entitled to remain informed with the latest updates as the study proceeds.

#### Additional consent provisions for collection and use of participant data and biological specimens {26b}

Not applicable. Consent for the collection and use of participant data is limited to this study.

### Interventions

#### Explanation for the choice of comparators {6b}

Participants are randomized on a 1:1 ratio into an experimental group and a control group. The experimental group receives NMES plus EMG biofeedback-mediated PFMT. The control group undertakes conventional PFMT [13] alone. Conventional PFMT is also included for the experimental group to ensure a sufficient frequency of PFMT, as 2–3 days a week of PFMT is often recommended for effective resistance training [23, 30]. The total frequency and time of PFMT are equal between groups.

#### Intervention description {11a}

As the latest guidelines for UI management recommend muscle training for at least 12 weeks, we set our intervention length as 12 weeks [23, 30].

In the baseline visit, all participants (regardless of group allocation as conventional PFMT is required for both) will receive a one-to-one education session. During this session, we brief them on the functional anatomy of PFM, assess their PFM contractive ability, and teach them the correct exercise technique. Timely feedback from the physiotherapist enhances their sensation of self-muscle activity and optimizes their training effectiveness by avoiding common mistakes, such as co-contraction of abdominal, deep hip, and thigh muscles. An individualized home-based PFMT program is prescribed for each according to their baseline PFM function. Evaluation and instruction are carried out in line with the PERFECT scheme (Power, Endurance, Repetitions, Fast, Every Contraction Timed). The initial resistance training program is as follows:

- Lift PFM upwards and inwards and squeeze the vagina, urethral and anal sphincters, as hard as they can without disturbing their breathing rhythm
- Hold it for 3–5 s before relaxing the muscles gently for the same time interval (3–5 s). Depending on individual ability, the time interval usually starts from 3 s and progresses to 5 s 4 weeks after and 5 s by the end of the intervention
- Repeat the sequence ten times in one set and perform three sets a day

During the intervention period, frequency measured as times per week differs between groups, as conventional PFMT mainly serves as a supplement to instrumented PFMT for the experimental group. We recommend the same frequency of 3 days a week after the intervention period ends.

After the baseline visit, women in both groups will also get an electronic or printed manual with their personalized PFMT program, emergency contact, and lifestyle advice, such as avoidance of heavy lifting besides breastfeeding and maintenance of unobstructed defecation.

In the subsequent appointments, participants are asked to perform their current program, allowing the physiotherapist to adjust the program according to their progress. Once their PFM strength hits grade 3 in the Modified Oxford Scale, the Knack maneuver will be added. That is pre-contracting PFM against increases in intra-abdominal pressure, represented by coughs in practice. A total of 5 appointments, at the weeks 0, 1, 4, 8, and 12 respectively, will be offered for both groups over the 12-week intervention period.

#### Control group: conventional PFMT

Participants assigned to the conventional PFMT group will perform the prescribed program 3 days a week for the entire intervention period. After each self-managed home-based PFMT session, participants are required to upload a record (note), detailing their contraction and relaxation time for each contractive sequence, repetition of sequences and sets, onto a system checked and responded by physiotherapists on a daily basis. Such recording improves adherence to the intervention, and thereby the control group would acquire the same amount of contact and supervision from medical staff with the experimental group.

#### Experimental group: NMES plus EMG biofeedback

NMES and EMG biofeedback will be performed 24 sessions, two weekly sessions of 30 min with MyoTrac Infinti Encoder (Model SA9800, Thought Technology Ltd., Canada) equipment, which contains two channels for EMG collection, channel A for PFM and channel B for rectus abdominis. NMES is delivered through electrodes on the RAYEE-A Vaginal Probe (Nanjing Vishee Medical Technology Ltd., China). Muscle electrophysiology is collected through electrodes on the same probe at a 14-bit resolution and a 2048-Hz sampling rate. The electric parameters are current type: functional electrical stimulation; frequency: 30–50 Hz; pulse duration: 300–500 μs; time: 10 min; 4–10s cycles; rise: 2–5 s fall: 2–5 s; and stimulation intensity: maximal level tolerable [7, 16]. Participants will be instructed to not actively contract pelvic floor muscles during the electrical stimulation and then perform PFMT following the visual instruction for 15 min. Two NMES and EMG biofeedback-mediated PFMT programs and one conventional PFMT program (see the above section) per week (with 3 PFMTs dispersed in 3 separate days) are arranged for the experimental group to ensure the same total amount of pelvic floor muscle training between two groups.

### Criteria for discontinuing or modifying allocated interventions {11b}

Interventions are suspended when (1) adverse events or other unintended responses occur, (2) more than three procedures and follow-ups are delayed, (3) information is so incompletely collected that it may affect the acquisition and interpretation of the results, or (4) severe protocol deviation and execution error happen. Women intending pregnancy or getting pregnant halfway will no longer receive the intervention. Participants can opt out if they plan on POP surgery. We will meet the participant’s request on discontinuation or group reversal to conventional PFMT. According to their PFM performance, training programs are modified to meet their needs in follow-up appointments.

### Strategies to improve adherence to interventions {11c}

Education on the roles pelvic support plays in POP as well as the impact of its progression on individual lives will be incorporated into the postpartum clinic and research appointments. With controlled training by NMES in the hospital scene and visualization of performance through EMG biofeedback, instrumented PFMT is theoretically motivative in nature. Encouragement from professionals can also raise participants’ self-efficacy. Thus, adherence to conventional PFMT could be enhanced by the timely response to their training records. Monthly calls will enable researchers to monitor their obedience and meanwhile collect encountered obstacles to hopefully overcome by optimizing study design.

### Relevant concomitant care permitted or prohibited during the trial {11d}

Usual postnatal care will continue during the trial.

### Provisions for post-trial care {30}

Usual postnatal care will be maintained after the trial.

### Outcomes {12}

See the “Plans for assessment and collection of outcomes {18}” section for a description on the clinical relevance of each outcome.

#### Primary outcomes

The primary outcome measure is:

- The proportion of participants with the improvement of at least one POP-Q stage compared to the baseline data

The primary outcome measure is calculated with each follow-up (M0PI, M6PI, M12PI, M24PI, M36PI, M stands for month, PI stands for post-intervention).

#### Secondary outcomes

The secondary outcome measures include:

- The proportion of participants suffering from each symptom at the time of follow-ups
- The average decreases in PFDI-20 score, PFIQ-7 score, IIQ7 score, and ED-5Q score and the average increase in PISQ-12 respectively from the baseline
- The average grade of PFM strength at the time of follow-ups
- The average EMG values in the pre-baseline, flick contraction, tonic contraction, endurance contraction, and post-baseline phases respectively at the time of follow-ups
- The average value in the diameter of levator hiatus at the time of follow-ups

The secondary outcome measures are calculated with each follow-up (M0PI, M6PI, M12PI, M24PI, M36PI).

### Participant timeline {13}

**Fig. 2:**
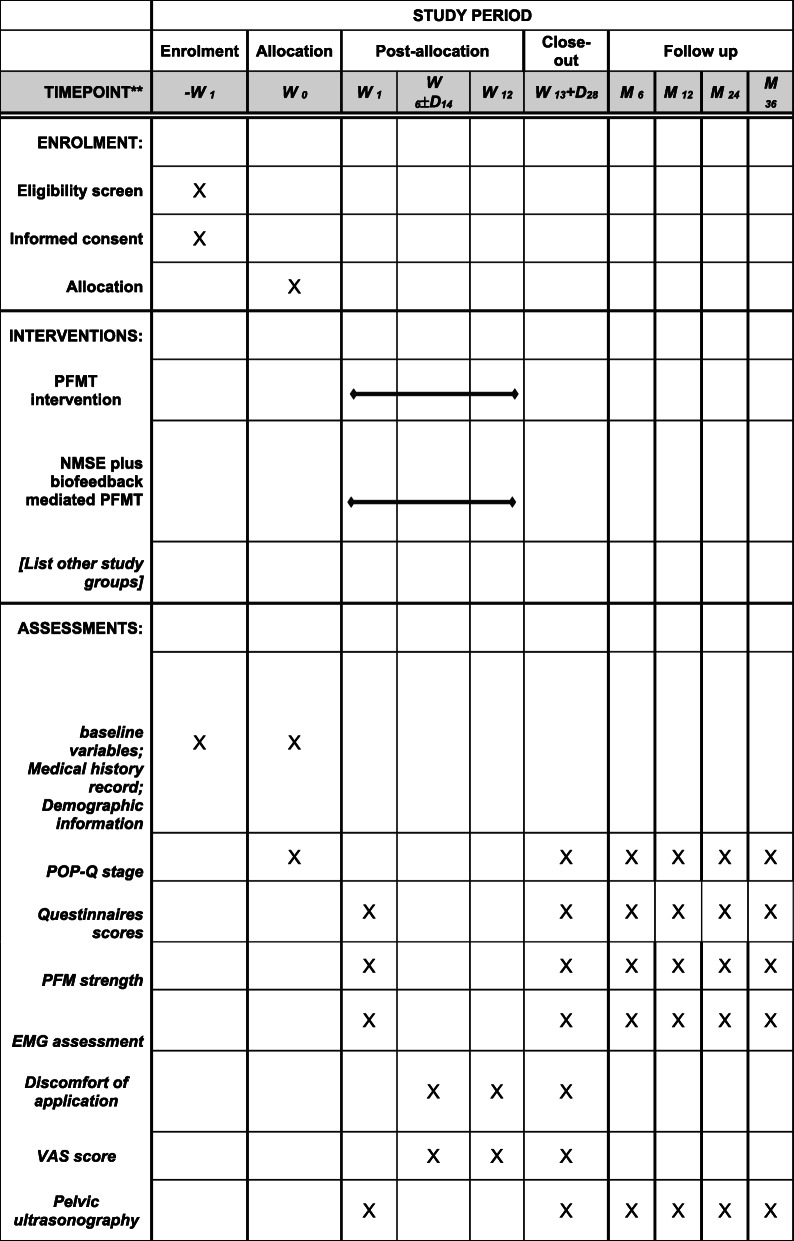
Content for the schedule of enrolment, interventions, and assessments

See Fig. 2 for the schematic diagram.

### Sample size {14}

Previous research has reported that 43.68~62.8% [31–33] of participants in the electrical stimulation plus PFMT group showed improvement in POP-Q staging while 15.4~20.9% [31–34] showed improvement in the PFMT group. Based on early-stage data from the ongoing pilot study, we estimate the proportion of participants with improved POP-Q stage to be 50% in the experimental group and 20% in the control group. Participants are randomized at a 1:1 ratio to the intervention and control groups. We applied a 2-sample superiority test on the proportions to calculate the sample size by PASS software. Based on our clinical treatment experience, we set the testing margin as 15% which indicates the significant clinical effectiveness. Assuming an attrition rate of 20% by the 36-month follow-up, a sample size of 284 women (142 in each arm) is required in the study to provide more than 80% power at a one-sided significance level of 0.05 for the 2-sample superiority test.

### Assignment of interventions: allocation

#### Sequence generation {16a}

Following baseline examinations, participants will be randomly assigned to the experimental group or the control group (1:1 ratio). A random number sequence with a size of 274 and a planned seed number of 2 is generated using the SAS9.4 system.

#### Concealment mechanism {16b}

The group allocation according to the random sequence is sealed in opaque envelopes, which is blinded to participants and research staff. New participants are sequentially numbered based on the chronological order of enrolment. Only then (after verification of eligibility, informed consent, and registration of enrolment) are they allowed to unwrap the corresponding envelope.

#### Implementation {16c}

The allocation sequence is generated by a registered clinical trial unit in China that is independent and password-protected from the investigators. Their employees are specialized in statistics and uninvolved in the following process. The principal investigator in each center is responsible for the eligibility verification, informed consent implementation, and conduct of allocation revealment during the enrolment process.

### Assignment of interventions: blinding

#### Who will be blinded {17a}

Due to the nature of the intervention, blinding of participants and physiotherapists delivering interventions or entering data is not possible but the research staff that carry out pelvic floor assessment, data coordinators, and analysts will be blinded to the group allocation result.

#### Procedure for unblinding if needed {17b}

Unblinding is not applicable.

### Data collection and management

#### Plans for assessment and collection of outcomes {18a}

Except for the past medical history that is only collected before and during the baseline visit, we will perform the rest of the items listed below in the baseline visit and follow-ups scheduled at and 6, 12, 24, and 36 months after the end of the intervention, to acquire primary and secondary outcome measures.

- Past medical history. We collect the gestation and delivery information for a baseline from the hospital’s Electronic Medical Record System. The important prognostic covariates for the clinical effectiveness, including newborn weight, the length of first/second stage, infant head circumference, and maternal body mass index, will be recorded.
- Lifestyle information: the habits of smoking or drinking, and the frequency of moderate-intensity exercise, which is important covariates for the long-term effectiveness. POP-Q stage. Anatomical severity of pelvic organ descent is measured and staged in a POP-Q examination during each follow-up appointment.
- Prolapse symptoms. We document the presence of POP-related symptoms among participants in each follow-up appointment. Symptoms selected for inquiry are those frequently included in the validated questionnaires and reported by POP patients.
- Questionnaires. POP-related urinary, colorectal-anal, and pelvic symptoms are evaluated with Pelvic Floor Distress Inventory-Short Form 20 (PFDI-20) and sexual symptoms using Pelvic Organ Prolapse/Urinary Incontinence Sexual Questionnaire (PISQ-12). The impact of such symptoms on quality of life is assessed by the Pelvic floor Impact Questionnaire-Short Form 7 (PFIQ-7), Incontinence Impact Questionnaire-Short Form 7 (IIQ-7), and EuroQol Five Dimensions Questionnaire (EQ-5D). These questionnaires are instruments with evidence of validity, reliability, and responsiveness and have been translated into Chinese. Participants are referred to complete these questionnaires at the end of each follow-up appointment.
- PFM strength grade. PFM strength that reflects the muscle function is graded using the Modified Oxford Scale (MOS) in digital palpation during each follow-up appointment.
- PFM electromyography. Glazer Protocol consists of PFM EMG values in the pre-baseline, flick contraction, tonic contraction, endurance contraction, and post-baseline phases. Obtained in each follow-up, these EMG values indicate muscle activity from an electro-physiological perspective objectively.
- Pelvic ultrasonography. Three-dimensional ultrasonography is widely used to assess the morphology of the pelvic floor muscles and pelvic organs, including the diameter of levator hiatus and position of the bladder and rectum at rest and during maximum Valsalva.

To promote data quality, all the assessments are done independently by two physiotherapists who have been given sufficient training. Altogether, these examinations comprehensively evaluate the morphology and function of PFM as well as the anatomical and clinical evidence of POP severity. Data collection forms are attached in the protocol.

- Economic analysis (cost-effectiveness analysis). The primary analysis will be undertaken alongside the trial using recommended methods [35, 36]. All costs and outcomes beyond 1 year will be discounted at 3.0% [37]. Incremental cost-effectiveness ratios (ICERs) will be computed by comparing the costs and effectiveness under the interventions of each group. The difference in effectiveness will be measured as the change in the proportion of the patients showing improvement of at least 1 level of POP-Q staging. The difference in cost-utility between the two groups will be expressed in terms of quality-adjusted life years (QALYs) that is calculated from the EQ-5D scores [36] using the actual time of follow-up. Multiple imputation is used to replace missing data. Analyses of total costs and QALYs use adjustments for baseline covariates via seemingly unrelated regression, and the final data is used to plot data on the cost-effectiveness plane.

#### Plans to promote participant retention and complete follow-up {18b}

Measures to help participants cling to the established schedule include:

- Seven days prior to their scheduled appointments, participants will receive a reminder message or email where they are notified of the accurate time, place, and recommended preparations for each procedure performed in that duration and they are asked to confirm their attendance. Those who do not reply to the message will receive a phone call.
- If participants report difficulties in arriving for follow-ups, shortage of time, or inconvenient traffic for example, we will try our best to give assistance, such as adjusting their schedule as long as the time window for each follow-up is not exceeded.
- We will contact participants who forget to return their questionnaires or miss other examinations within a week to see if they need reminders or other forms of help in the future.

If for any reason they opt to withdraw, no more intervention will be conducted, and no more data will be collected. Data collected before their dropout is used for further analysis under their consent. They will be invited to complete a questionnaire on the reasons for quitting.

#### Plans for collection, laboratory evaluation, and storage of biological specimens for genetic or molecular analysis in this trial/future use {33}

Not applicable as no biological specimens are collected.

#### Plans to give access to the full protocol, participant-level data, and statistical code {31c}

Access to the full protocol, participant-level data, and statistical code will be given by the corresponding author upon reasonable request.

### Data management {19}

Upon enrolment, each participant will be assigned a unique study ID for substitute of their identity information, the only form of participant referral that will be presented on case report forms, used for statistical analysis, and provided for publication.

We will hire a Clinical Research Coordinator and Clinical Research Associate to help with data management. They are obliged to sign a confidentiality agreement before stepping in. Under the supervision of the principal investigator, they fill the case record forms and upload data onto the Epidata. Data are kept in a secure storage that only authorized staff are granted access by the principal investigator.

### Confidentiality {27}

Name and the phone number of potential participants are registered during the routine postpartum consultation if they express interest after reading the recruitment advertisement. Contact information is accessed for the PROSPECT study only by the principal investigator. Upon enrolment, more detailed identity information will be asked merely for legality reviews by national regulatory authorities and Beijing Hospital Ethics Committee and other involved clinical institutions. All personal information mentioned above will be kept in rigorous confidentiality in conformity to laws and regulations. Documentation of this personal information will be permanently locked after the trial.

### Statistical methods

#### Statistical methods for primary and secondary outcomes {20a}

All statistical analyses will be carried out according to the Statistical Analysis Plan and Health Economic Analysis Plan. The primary analysis will be performed at the time right after intervention and at the end of the trial when a 36-month follow-up has been completed. The independent data monitoring and ethics committee (DMEC) will review confidential interim analyses of accumulating data at its discretion, at least annually. The primary effectiveness analysis will be based on the intention-to-treat (ITT) principle.

In the analysis of the primary outcome, we will use the chi-square test to compare the proportion of participants showing improvement of at least 1 level of POP-Q staging among experimental and control groups right after intervention. Furthermore, a binary logistic regression model will be adopted to adjust potential covariate variables like the newborn weight, the length of first/second stage, infant head circumference and the maternal body mass index, etc. For analysis of secondary outcomes, we used appropriate models: the multilevel mixed models for continuous outcomes (PFDI-20, PFIQ-7, PISQ-12, and ICIQ SF-7 scale scores, pelvic floor muscle electromyography values, VAS and QALY of each incremental cost) for the repeated measurements at 6, 12, 24, and 36 months, chi-square test and logistic regression for categorical outcomes (POP-Q staging, pelvic floor muscle strength). The safety index (AE incidence) was analyzed with the chi-square test.

#### Interim analyses {21b}

A single primary analysis will be performed. No interim analysis is planned in the current study.

#### Methods for additional analyses (e.g., subgroup analyses) {20b}

Subgroup analyses will be performed within the following groups: age (< 35/≥35 years) and additional moderate-intensity exercise (Y/N). The statistical significance level will be set at 5%. Heterogeneity of treatment effects among subgroups will be tested by interactive subgroup analysis.

#### Methods in analysis to handle protocol non-adherence and any statistical methods to handle missing data {20c}

Non-adherence will be processed by an intention-to-treat method in the primary analysis. Missing data of primary outcome will take baseline data for reference and will be further assessed in sensitivity analyses. Due to the randomized design, multivariable models will not be used to adjust for confounding, and therefore, the absence of covariates will not affect the primary analysis.

### Oversight and monitoring

#### Composition of the coordinating center {5d}

The principal investigator leading all coordinating centers designed this trial. For each center, a principal investigator is delegated respectively. They are responsible for the refinement of the protocol, the CRF, and other materials. Personnel-wise, they construct a steering team (see the section below) and recruit staff to run the trial. They organize weekly and monthly meetings for quality control. All staff convened to the study, including PI themselves and other healthcare providers, research coordinators, and data analysts, will contribute to the smooth operation of the whole process.

#### Composition of the trial steering committee and data monitoring committee {5d, 21a, 23} and frequency and plans for auditing trial conduct {23}

Oversight on safety, data quality, and financial management will be provided by the Ethics Committee and the Clinical Trial Research Center in the academic hospitals, as well as the staff in the PROSPECT study. The study protocol undergoes reviews and approval by the hospital Ethics Committee prior to its launch. Any modification to the protocol should be submitted and approved before continuation of the study. The Ethics Committee also demands principal investigators to hand in research progress reports. Audits to monitor data management are carried out by the quality assurance (QA) department of Clinical Trial Research Center once a year, where investigators of the study are masked. Advice is returned to PIs and their revision will be followed in later meets. These committees are mainly based on hospitals. Funders are not implicated in the trial design and conduct, nor are they involved in the supervision. No competing interests are present concerning this study.

#### Adverse event reporting and harms {22}

Adverse events are defined as worsening of existing POP or newly developed symptoms related to PFDs while serious adverse events are defined as affecting normal daily life. As extensively used treatments in clinical practice, side effects and adverse events of PFMT and NMES are scarcely observed. However, some patients do feel pain or discomfort with NMES. Adverse events of vaginal symptoms, back and abdominal pain, short of breath, and chest pain have been reported in POP patients treated with PFMT although considered irrelevant to the intervention. For safety concerns, participants assigned PFMT with NMES and EMG biofeedback device are all called to the hospital settings for its use, allowing us to closely monitor complications. Whatever unexpected is experienced by the participants, we will assess (1) whether it has been already identified, (2) whether it is related to the intervention procedure, and (3) whether it subsides after the cessation of the intervention. Regardless of the severity, such events are recorded on the Adverse Events Report Form with the time of onset, seriousness, duration, measures undertaken, and results. The forms will soon be transferred to the principal investigator and the Ethics Committee.

#### Plans for communicating important protocol amendments to relevant parties (e.g., trial participants, ethical committees) {25}

According to hospital regulations, any protocol amendments agreed by all investigators in the study should be sent to the Ethics Committee for reviews. Principal investigators need to attend the following ethnic meetings to discuss their adjustments. No further procedures are allowed before approval. Notification of approved modifications will be forwarded to enrolled participants. We will update our registration upon approval from our steering committee.

#### Dissemination plans {31a}

Results of this study will be presented in conference and published in scientific papers. In a we-media era when WeChat posts often achieve immense public attention in China, we plan to create a WeChat Official Account to popularize the benefits of pelvic floor rehabilitation with evidence established in our study.

## Discussion

With the aging population, the incidence of pelvic organ prolapse is on a rise. Also, pelvic organ prolapse is always concomitant with other PFDs, which caused heavy health economic and social burden for the increasing demand of medical care. The injury of pelvic floor muscles and connective structure during childbirth is a major risk factor for PFDs. Pelvic floor rehabilitation under professional guidance, with the goal of promoting the recovery of damaged nerves and muscles, is essential in the prevention and treatment of PFDs. The postpartum PFMT is considered to not only significantly reduce the incidence of pelvic floor dysfunction during 6–12 months postpartum [38, 39], but also possibly reduce the dysfunction due to anatomical changes and aging. There is clear evidence that urinary and fecal incontinence can benefit from PFMT. The American Congress of Obstetricians and Gynecologists (ACOG) Clinical Practice Guidelines for Pelvic Organ Prolapse (2007) recommend pelvic floor muscle training as an adjuvant treatment for symptomatic prolapse [40]. However, the evidence on the short- and long-term effectiveness of PFMT and biofeedback methods to treat prolapse is scarce. Therefore, the PROSPECT study, which evaluates pelvic floor muscle function in terms of POP-Q staging, quality of life questionnaires, and the potential impact on current and future public health economics, aims to provide such evidence. A particular advantage of the PROSPECT trial is that it guarantees all participants the access to pelvic floor rehabilitation. This study hopefully helps to test whether instrument-based biofeedback-mediated PFMT is more effective and cost-effective than self-management. If the PROSPECT study arrives at such conclusions, we hope to add more value and confidence to the popularization of NMES and biofeedback approach.

## Trial status

The internal pilot of the PROSPECT trial in Beijing Hospital (the first center of this study) has been approved by Ethics Committee and registered on the Chinese Clinical Trial Registry (ChiCTR2100021719). Its recruitment has begun in July 2020 and will terminate by December 2024. A total of 75 patients have been enrolled in our center (Beijing Hospital) and statistical analysis of their data is ongoing. Applications of other centers are underway. The current protocol has been refined on version 2 (approved on February 28, 2019). We apply the Standard Protocol Items: Recommendations for Interventional Trials (SPIRIT) to structure our protocol.

## Abbreviations

PROSPECT: To treat *pro*lapse *s*uffered *p*ostpartum with *e*lectrical stimulation-*c*ombined *t*raining of pelvic floor muscle
PFM: Pelvic floor muscle
PFMT: Pelvic floor muscle training
NMES: Neuromuscular electrical stimulation
PFD: Pelvic floor dysfunction
POP: Pelvic organ prolapse
UI: Urinary incontinence
POP-Q: Pelvic Organ Prolapse Quantification System
EMG: Electromyography
DMEC: Data monitoring and ethics committee
ITT: Intention-to-treat
